# Editorial: Precision vaccinology for infectious diseases

**DOI:** 10.3389/fimmu.2024.1400443

**Published:** 2024-04-18

**Authors:** Mahbuba Rahman, Mehdi Adeli, Herb E. Schellhorn, Puthen Veettil Jithesh, Ofer Levy

**Affiliations:** ^1^ Department of Biology, McMaster University, Hamilton, ON, Canada; ^2^ Department of Pediatrics, Hamad Medical Corporation, Doha, Qatar; ^3^ College of Health & Life Sciences, Hamad Bin Khalifa University, Doha, Qatar; ^4^ Precision Vaccines Program, Department of Pediatrics, Boston Children’s Hospital, Boston, MA, United States; ^5^ Harvard Medical School, Boston, MA, United States; ^6^ Broad Institute of Massachusetts Institute of Technology (MIT) & Harvard, Cambridge, MA, United States

**Keywords:** precision vaccinology, infectious diseases, global health, immunity, vulnerable populations

In the ever-evolving landscape of infectious diseases, the quest for effective preventive measures has reached a pivotal stage. The rise of precision vaccinology signifies a groundbreaking shift in vaccination strategies, offering bespoke solutions that acknowledge the diversity of individual immune responses. This Research Topic of *Frontiers in Immunology* explored precision vaccinology, where distinct immunity in diverse vulnerable populations dynamic interplay of genes, proteins, and metabolites within the human body is leveraged to craft precise and potent vaccines against infectious pathogens. International researchers have joined forces to collaboratively advance precision vaccinology. Through original research, comprehensive reviews, and meticulously curated datasets, these esteemed experts shed light on the current challenges and opportunities in vaccine discovery and development, guiding the way towards immunization approaches tailored to meet the specific requirements of particular groups.

Aiman et al. introduced a novel approach to counter the emerging threat of monkeypox virus (MPXV), that bears similarities to smallpox. Employing immune-informatics, they identified potential vaccine targets to enhance immunity against MPXV, employing diverse analyses to pinpoint three outer membrane and extracellular proteins. These selections were made based on criteria such as antigenicity and allergenicity, ensuring broad protection against various MPXV strains worldwide. They designed multi-epitope vaccine constructs, incorporating nine overlapping B-cell and T-cell epitopes coupled with suitable adjuvants to augment immune responses. Via molecular modeling and structural validation, the quality of the vaccine constructs was confirmed, with MPXV-V2 displaying promise for further exploration. This research lays a solid foundation for the development of novel safe and effective MPXV vaccines.

Li et al. addressed the urgent challenge of duck cholera, a serious threat to the duck industry caused by *Pasteurella multocida*. Concentrating on the prevalent Type A serotype, they successfully cloned and expressed VacJ, PlpE, and OmpH proteins from *P. multocida* PMWSG-4 in *E. coli*. These engineered proteins, along with a single-phase water-in-oil adjuvants, triggered substantial antibody responses in vaccinated ducks. The adjuvant was developed by Guangdong Wen’s Foodstuff Group Co. Ltd. Li et al. observed that the vaccine formulations exhibited varying levels of protection against *P. multocida* A:1, suggesting the potential of using recombinant PlpE or OmpH fusion proteins in combination with adjuvants to effectively combat avian cholera.

Lu et al. delved into the complexities surrounding the administration of viral respiratory vaccines, aiming to address uncertainties regarding impact of co-administration of vaccines on safety immunogenicity. Their meta-analysis of data from PubMed, Embase, Cochrane Central Register of Clinical Trials, Web of Science, WHO COVID-19 Research, and ClinicalTrials.gov databases assessed variables affecting rates of adverse events as well as efficacy. They analyzed randomized controlled trials involving adults who received co-administered viral respiratory vaccines alongside others, revealing nuanced effects on seroconversion and seroprotection rates across vaccine groups. By emphasizing factors such as vaccine type, adjuvant content, and recipient demographics, their study underscored the need for tailored precision vaccination strategies to optimize safety and efficacy.

Qassim et al. explored the infectivity of SARS-CoV-2 infections in Qatar throughout 2021, characterized by the sequential predominance of Alpha, Beta, and Delta variants. Analyzing 18,355 RT-qPCR-genotyped infections, they correlated cycle threshold (Ct) values with indicators of infectivity. Alpha and Delta variants exhibited notably lower Ct cycles by 2.56 and 4.92, respectively, compared to Beta variants. Ct values were high, particularly in children under 10, suggesting reduced infectivity. Vaccinated individuals displayed higher Ct values, which declined post-second dose. Pre-existing immunity from vaccination or prior infection decreased infectivity, albeit diminishing over time post-vaccination. Notably, the Delta variant exhibited heightened infectivity. These findings underscore the role of vaccination in reducing infectivity while highlighting variant-specific risks.

Papukashvili et al. delineated a strategic framework for developing nucleic acid-based universal monkeypox (MPX) vaccine candidates. Despite historical neglect, MPX has gained global attention for its spread beyond African borders, particularly impacting Central and West African nations. Given the absence of smallpox vaccination in most infected individuals, preventive measures are crucial, underscoring the importance of vaccination. Nucleic acid vaccines, particularly mRNA and DNA platforms, hold promise, capitalizing on the success of COVID-19 mRNA vaccines. Their cost-effectiveness and rapid development potential offer significant advantages. The review stresses the urgent need for universal MPXV vaccines and explores strategies for expedited nucleic acid vaccine development, providing a promising path to mitigate the global MPXV threat.

Yousaf et al. undertook immuno-informatics profiling of the MPXV cell surface binding protein to design an advanced multi-valent peptide-based vaccine. Amidst the escalating global challenge posed by monkeypox viral infection, multi-valent peptide vaccines offer promising solutions by eliciting both cell-mediated and humoral immune responses. Employing subtractive proteomics and reverse vaccinology, they craft a novel, *in silico* peptide-based vaccine construct. Through rigorous analysis, they pinpoint highly antigenic, virulent epitopes with wide population coverage. Molecular docking studies reveal robust binding affinity with immune receptors, suggesting potent induction of immune responses. While experimental validation remains imperative, their findings suggest potential therapeutic efficacy against monkeypox, underscoring the necessity for further investigation.

Li et al. conducted a preliminary genome-wide association study (GWAS) to explore the genetic factors influencing the antibody response to inactivated SARS-CoV-2 vaccines. Despite the crucial role of vaccines in combating the COVID-19 pandemic, understanding individual variations in immune response remains limited. By analyzing data from 168 vaccine recipients, they identified 177 single nucleotide polymorphisms (SNPs) across 41 independent loci associated with IgG, total antibodies, or neutral antibodies. Notably, the rs4543780 variant within the FAM89A gene demonstrated a correlation with total antibody levels and was implicated as a potential regulatory variant affecting FAM89A gene expression. These findings offer insights into the molecular mechanisms shaping SARS-CoV-2 vaccine immunogenicity, providing a basis for further research in this field.

Ghorbani et al. explored the relationship between oral microbiome variation and salivary antibody responses post-COVID-19 vaccination in healthy individuals and those living with HIV (PLHIV). Analyzing participants from the COVAXID trial who received the BNT162b2 mRNA vaccine (n=115), they assessed saliva and serum antibody levels over six months alongside individual oral microbiome diversity. High versus low vaccine responders were compared, revealing distinct microbiome features. Low responders exhibited an abundance of Gram-negative, anaerobic species, while high responders had predominantly Gram-positive, saccharolytic facultative anaerobes. Classifier analysis supported the oral microbiome’s influence on vaccination outcomes, suggesting microbiome-targeted interventions for enhancing the durability of mucosal vaccine immunogenicity.

Goll et al. introduced the Vacc-SeqQC project, which aims to assess RNA sequencing (RNA-seq) in clinical vaccine studies. They utilized longitudinal samples of peripheral blood mononuclear cells (PBMCs) from a trial of a live attenuated *Francisella tularensis* vaccine, comparing RNA-seq data from two different sites. The study evaluated various factors including gene filtering, external RNA controls, fold change cutoffs, read length, and sequencing depth. Their findings suggest that filtering out low-expression genes improves accuracy and consistency between sites. Read length had minimal impact on detecting differential gene expression, while fold-change cutoffs reduced agreement. Additionally, the study highlights the importance of sample size and effect size in determining statistical power. Overall, the Vacc-SeqQC project provides valuable benchmarks and guidelines for future vaccine studies utilizing RNA-seq technology.

Armero-Gimenez et al. introduced a swift screening and production technique for generating immunogenic Virus-Like Particles (VLPs) utilizing a tobacco BY-2 cell-free protein synthesis (CFPS) system. VLPs are an attractive vaccine platform due to their robust immunogenicity, stability, and safety characteristics. The research showcases the efficient production of hepatitis B core (HBc) carrier VLP variants in the BYL system, enabling thorough characterization. Scaling up to 1L in batch mode resulted in substantial quantities of native HBc VLPs within 48 hours, maintaining high yield. Immunogenicity assessments confirmed rapid recognition by dendritic cells and cytokine production in human cells, underscoring the potential of BYL for swift VLP screening and manufacturing.

Chen et al. investigated precision-engineering techniques tailored for subunit vaccine particles to enhance protection against infectious diseases. While protein subunit vaccines offer safety advantages compared to whole-cell vaccines, they often face challenges with lower immunogenicity. Particulate subunit vaccines, however, demonstrate enhanced efficacy by provoking robust immune responses, thereby conferring protective immunity. Ensuring proper antigen conformation and functionality is vital for inducing an effective immune response, yet production obstacles persist, particularly in bacterial hosts. The study explores strategies to overcome these challenges, including innovative assembly methods employing both non-covalent (e.g., biotin-avidin affinity) and covalent (e.g., SpyCatcher-SpyTag) attachments for complex antigens with multiple post-translational modifications. These approaches aim to optimize both humoral and cellular immune responses, advancing the development of more potent subunit vaccines.

Jiao et al. conducted an analysis focusing on the conserved protective epitopes present on the hemagglutinin (HA) protein of influenza A viruses. These epitopes play a pivotal role in the development of universal influenza vaccines and targeted therapeutic interventions. Through the identification of broadly neutralizing antibodies (bnAbs) that target HA over the past 15 years, researchers have delineated binding epitopes, providing novel insights into conserved protective epitopes. The review compiles data on >70 bnAb antigenic epitopes, emphasizing five crucial regions on HA: the hydrophobic groove, receptor-binding site, occluded epitope region, fusion peptide region, and vestigial esterase subdomain. This comprehensive analysis offers valuable targets for the design of effective vaccines and therapeutics against influenza A virus infections.

Radwanska et al.introduced the macrophage infectivity potentiator protein (TcMIP) derived from *Trypanosoma cruzi* as an innovative pro-type 1 immunostimulatory factor for neonatal human cells and a potential vaccine adjuvant in mice. *In vitro*, recombinant TcMIP (rTcMIP) induced production of CCL2 and CCL3 in umbilical cord blood cells from healthy newborns after 24 hours of incubation, followed by IFN-γ secretion after 72 hours with IL-2 and IL-18 supplementation. *In vivo* experiments in neonatal mouse immunized model demonstrate that rTcMIP act as adjuvant and enhances IgG antibody responses to diverse antigens, promoting Th-1-dependent IgG2a isotype production without exacerbating IgE responses. These findings suggest the potential of rTcMIP as a neonatal vaccine adjuvant, offering a promising avenue to effectively combat early-life infections.

Anwardeen et al. conducted a retrospective study examining the metabolic effects of BCG vaccination in COVID-19 patients with type 2 diabetes (T2D). By comparing BCG-vaccinated and non-vaccinated individuals, the study identifies distinct metabolomic profiles. While BCG vaccination appears to offer protection in non-diabetic patients, it correlates with severe COVID-19 symptoms in those with T2D. Specifically, BCG-vaccinated T2D patients exhibit elevated levels of sarcosine, cholesterol esters, and aconitic acid, whereas non-T2D counterparts show increased levels of spermidine and glycosylceramides. Notably, sarcosine synthesis decreases in BCG-vaccinated T2D patients, potentially compromising viral antigen removal. These findings underscore a complex metabolic response to BCG vaccination in T2D COVID-19 patients, highlighting the need for further investigation into its implications.

Borghi et al. delved into exploring different arrangements of the SARS-CoV-2 spike protein delivered through integrase-defective lentiviral vectors (IDLVs) to elicit enduring functional immune responses with distinct immunogenicity profiles. Utilizing a mouse model, they evaluated IDLVs harboring spike protein variants with various alterations, including prefusion-stabilizing double proline substitutions, mutations in the furin cleavage site, D614G mutation, and cytoplasmic tail truncation, targeting multiple SARS-CoV-2 variants. Robust and persistent production of anti-receptor binding domain binding antibodies, neutralizing antibodies, and T cell responses were observed lasting six months after immunization. Particularly, the IDLV delivering Spike with combined modifications demonstrated superior T cell immunity and sustained antibody levels, effectively neutralizing diverse variants with the exception of Omicron variants.

Lorenz et al. examined the humoral immune response targeting specific SARS-CoV-2 Spike protein epitopes within cohorts vaccinated with nucleic acid and protein-based vaccines. A linear B-cell epitope adjacent to the furin cleavage site in the S1 domain was identified, revealing distinctive recognition patterns between vaccine groups. Plasma samples from patients notably exhibited recognition of epitopes within the fusion peptide and connector domain of Spike S2, known for their ability to impede viral infection. Notably, among vaccine recipients, amino acids 657-671, situated close to the furin cleavage site, induced a greater antibody response in AZD1222 and BNT162b2 cohorts compared to NVX-CoV2373 recipients. These insights into antibody function within this region contribute valuable information for the design of future vaccines targeting SARS-CoV-2.

Djebbara et al. delved into investigating the immunostimulatory properties of the macrophage infectivity potentiator (rTcMIP) derived from *Trypanosoma cruzi*. Their study revealed that rTcMIP triggers the production of IFN-γ and TNF-α in both neonatal and adult blood cells, thereby enhancing type 1 adaptive immune responses. Through engagement with Toll-like receptors 1/2 and 4 (TLR1/2 and TLR4), rTcMIP activates the MyD88 pathway, inducing IFN-γ in natural killer (NK) cells and TNF-α in monocytes and dendritic cells. Notably, TNF-α was found to augment IFN-γ expression. Although neonatal cells exhibited lower responses compared to adult cells, these findings suggest the potential of rTcMIP as an adjuvant for vaccines targeting both early-life and adult populations, thereby bolstering T and B cell immunity.

Purcell et al. delved into the discrepancy between vaccine efficacy observed in clinical trials and real-world effectiveness, particularly among vulnerable populations such as children, the elderly, and individuals with chronic conditions. They highlighted the need for tailored immunization schedules targeting these groups to optimize vaccine responses. While current vaccination approaches primarily consider basic clinical parameters, they often overlook the distinct nature of individual immune responses. The authors advocated for the development of precision vaccines capable of addressing variations in vaccine response influenced by factors like immunogenetics and baseline health status. They underscored the significance of polyfunctional antibodies, which play a dual role in neutralization and effector functions, in conferring vaccine-induced protection. Proposing a rational mechanistic approach to vaccine design, they advocated for enhancing public health outcomes by ensuring vaccines are tailored to individual immune profiles.

Beavis et al. explored the efficacy of combined intranasal and intramuscular vaccination approaches utilizing parainfluenza 5 (PIV5), simian adenovirus ChAdOx1, and poxvirus MVA vectors to amplify HIV-1-specific CD8+ T cell responses in mucosal tissues. Their study, conducted in BALB/c mice, reveals that incorporating PIV5 vectors expressing HIVconsvX immunogens into the ChAdOx1-MVA regimen results in synergistic enhancement of mucosal CD8+ T cell induction. Encouraged by phase 1 trial findings indicating the safety and immunogenicity of ChAdOx1 and MVA vaccines for HIV-1, and PIV5 vaccines for other respiratory viruses, the research suggests the potential benefits of combining these vectors to enhance immunization against HIV-1 and other viral pathogens.

Zhu et al. conducted a post-market cross-sectional study to evaluate antibody responses to an inactivated SARS-CoV-2 vaccine in individuals >50 years of age. Detectable but generally low levels of total serum SARS-CoV-2-specific antibodies were noted across all age groups. Antibody levels decline with age, with significant differences observed between age groups. The vaccine demonstrates effectiveness in older individuals, highlighting the importance of vaccination for this population. However, those with hypertension or diabetes show lower antibody responses. The study underscores the need for tailored vaccination strategies for vulnerable populations including older adults and those with chronic conditions, to optimize vaccine efficacy.

AbdelWareth et al.conducted a comparative paired analysis between naturally infected individuals and those with hybrid immunity (from infection and vaccination) to SARS-CoV-2. Antibody responses were measured in 197 male participants post-vaccination. A significant increase in trimeric spike, nucleocapsid, and ACE2-RBD blocking antibodies was observed after vaccination. The study highlights a robust immune response, especially in ACE2-RBD blocking antibodies, post-vaccination following natural infection. Notably, a positive dose-response relationship exists between vaccine doses and antibody concentration. This underscores the importance of vaccination in augmenting immunity, particularly in individuals with prior infection, for enhanced protection against SARS-CoV-2.

Li et al. conducted a review focusing on recent advancements in pneumococcal protein vaccines, aiming to address the persistent global health threat posed by pneumococcal infections. Despite the effectiveness of current polysaccharide and conjugate vaccines, their limited coverage and the emergence of non-vaccine serotypes emphasize the necessity for alternative approaches. Protein-based vaccines targeting conserved surface proteins of *Streptococcus pneumoniae* demonstrate considerable promise. The review delineates key protein vaccine candidates, their efficacy in animal models, and discusses existing challenges and future prospects. This comprehensive examination highlights the potential of protein-based pneumococcal vaccines in addressing the changing landscape of pneumococcal infections, while also identifying avenues for further investigation.

Yang et al. introduced a rapid *de novo* identification strategy for bacterial antigens derived from clinical isolates, essential in combatting emerging infectious diseases. Focusing on *Actinobacillus pleuropneumoniae*, a significant threat to the swine industry, they employed an integrated approach combining proteosurfaceomics, secretomics, and BacScan technologies. BacScan is a genome-wide tool identifying highly immunogenic proteins in bacteria using T7 phage displaying library. The integrated method led to the discovery of three previously unidentified protective proteins, notably HBS1_14, facilitating the development of a multivalent subunit vaccine. The innovative approach showcased by this study holds promise for expediting the development of antigen-matched vaccines against emerging bacterial pathogens, thereby providing a valuable tool in controlling the spread of novel infectious diseases across human and animal populations.

Collectively, these studies represent significant strides in precision vaccinology, offering insights into vaccine design, development, and deployment. By addressing key challenges and leveraging innovative approaches, researchers are paving the way for personalized immunization strategies that enhance protection against infectious diseases. As we navigate the complex landscape of global health threats, collaboration and innovation remain paramount in the pursuit of discovery, development, and optimization of effective vaccines for all.

## Author contributions

MR: Conceptualization, Writing – original draft, Writing – review & editing. MA: Writing – review & editing. HS: Writing – review & editing. PJ: Writing – review & editing. OL: Writing – review & editing.

